# Differences between men with screening-detected versus clinically diagnosed prostate cancers in the USA

**DOI:** 10.1186/1471-2407-5-27

**Published:** 2005-03-08

**Authors:** Richard M Hoffman, S Noell Stone, David Espey, Arnold L Potosky

**Affiliations:** 1 Medicine Service, New Mexico VA Health Care System, Albuquerque, New Mexico, USA; 2 New Mexico Tumor Registry, University of New Mexico Health Sciences Center, Albuquerque, New Mexico, USA; 3 Non-communicable Disease Epidemiology Unit, London School of Hygiene and Tropical Medicine, London, UK; 4 CDC Division of Cancer Prevention and Control and Indian Health Service National Epidemiology Program, Albuquerque, New Mexico, USA; 5 Division of Cancer Control and Population Sciences, National Cancer Institute, Bethesda, Maryland, USA

## Abstract

**Background:**

The advent of prostate specific antigen (PSA) testing in the United States of America (USA) has led to a dramatic increase in the incidence of prostate cancer in the United States as well as the number of men undergoing aggressive treatment with radical prostatectomy and radiation therapy. We compared patient characteristics and treatment selection between American men with screening-detected versus clinically diagnosed prostate cancers.

**Methods:**

We evaluated 3,173 men with prostate cancer in the USA. Surveys and medical records provided information on demographics, socioeconomic status, comorbidities, symptoms, tumor characteristics, and treatment. We classified men presenting with symptoms of advanced cancer – bone pain, weight loss, or hematuria – as "clinically diagnosed"; asymptomatic men and those with only lower urinary tract symptoms were considered "screening-detected." We used multivariate analyses to determine whether screening predicted receiving aggressive treatment for a clinically localized cancer.

**Results:**

We classified 11% of cancers as being clinically diagnosed. Men with screening-detected cancers were more often non-Hispanic white (77% vs. 65%, P < 0.01), younger (36% < 65 years vs. 25%, P ≤ 0.01), better educated (80% ≥ high school vs. 67%, P < 0.01), healthier (18% excellent health vs. 10%, P < 0.01), and diagnosed with localized disease (90% vs. 75%, P < 0.01). Men with screening-detected localized cancers more often underwent aggressive treatment, 76% vs. 70%, P = 0.05.

**Conclusion:**

Most cancers were detected by screening in this American cohort. Appropriately, younger, healthier men were more likely to be diagnosed by screening. Minority status and lower socio-economic status appeared to be screening barriers. Screening detected earlier-stage cancers and was associated with receiving aggressive treatment.

## Background

Prostate-specific antigen (PSA) testing was introduced in the United States of America (USA) in the late 1980s with Federal Drug Administration (FDA) approval for prostate cancer surveillance [1]. However, the test indications were soon expanded to include prostate cancer screening. By the early 1990s, the American Urologic Association and the American Cancer Society were recommending PSA testing, along with digital rectal examination (DRE), as part of annual prostate cancer screening [2,3]. The advent of PSA testing led to a dramatic increase in the incidence of prostate cancer in the USA, with the number of new cases rising from 152,811 in 1990 to over 230,000 in 1992 [4,5]. During the past decade, the number of American men undergoing aggressive treatment with radical prostatectomy and radiation therapy also increased substantially [6,7].

Urologic screening studies provide the most comprehensive information about the men undergoing PSA screening [8-10]. Several trials have taken place in both Europe and the USA. In general, study subjects usually were recruited through advertisements and they were screened with combinations of PSA, DRE, and transrectal ultrasound. The average age of these study participants was in the mid-60s, and minority subjects were not well represented. Minimal data were provided on symptoms, comorbidity, or socioeconomic status. Among American men diagnosed with clinically localized prostate cancers, approximately 90% underwent treatment with radical prostatectomy or radiation therapy.

Population-based data on PSA screening are largely unavailable, including information on the proportion of prostate cancers diagnosed by screening, the demographic, socioeconomic, and clinical characteristics of men with screening-detected cancers, and the association of screening detection with treatment decisions. We used data from the United States-based Prostate Cancer Outcomes Study (PCOS) to 1) determine the proportion of screening-detected prostate cancers in a population-based cohort, 2) compare baseline demographic, socioeconomic, and clinical characteristics between men with screening-detected versus clinically diagnosed cancers, and 3) determine whether men with screening-detected clinically-localized prostate cancers were more likely to undergo aggressive treatment (radical prostatectomy or radiation therapy).

## Methods

### Study population

The American National Cancer Institute instituted the PCOS in 1994 to measure practice patterns and health-related quality of life among men diagnosed with prostate cancer in the United States. Methods for this multi-site, longitudinal project are described elsewhere [11]. Briefly, PCOS subjects were men histologically diagnosed with prostate cancer between October 1, 1994 and October 31, 1995. Subjects were identified using a rapid case ascertainment system by the six participating National Cancer Institute Surveillance, Epidemiology and End Results (SEER) cancer registries (Atlanta, Georgia metropolitan area; Los Angeles County California; King County, Washington; Connecticut; Utah; and New Mexico). Eligible subjects were residents of the areas covered by these registries at the time of diagnosis and were between the ages of 39 and 89 years, except in King County, where only men over 60 years were eligible. The institutional review board of each participating institution approved the study.

Eligible patients were sampled within strata of age, race/ethnicity, and tumor registry to approximate a sample representative of the United States population of prostate cancer patients. The PCOS oversampled younger men and minorities and excluded patients with race/ethnicity other than non-Hispanic white, African American, or Hispanic, because their sample sizes were small.

A total of 11,137 men with prostate cancer comprised the eligible patient population for the study and the PCOS randomly selected 5,672 of these men. Among these selected patients, 3173 (55.9%) completed a health-related quality-of-life survey questionnaire 6 months after initial diagnosis. We used survey and medical record data collected from these subjects to evaluate differences in patient characteristics and treatments between men with screening-detected cancers and those who were diagnosed clinically. Responders to the PCOS survey were younger than non-responders and more likely to be non-Hispanic white and have a higher socioeconomic status. A substantial proportion of the responders had regional stage and moderately differentiated cancers, while non-responders had a greater proportion of distant stage and poorly differentiated cancers. Responders also were more likely to receive radical prostatectomy [11].

### Data collection

Investigators contacted eligible subjects by mail and/or telephone requesting them to sign a release form allowing review of all medical records from any physicians and facilities diagnosing and/or providing care for prostate cancer. Records were obtained from private and public hospitals, freestanding radiological or surgical centers, Veterans Administration hospitals, Health Maintenance Organizations, and private physician offices. Certified Tumor Registry abstractors collected baseline information on demographics, clinical symptoms before diagnosis (systemic and urinary), comorbidity, diagnostic procedures and results (including PSA levels and digital rectal examination findings), clinical staging, tumor characteristics, and treatment details. The PCOS re-abstracted a random sample of 5% of records to assess and correct any systematic coding errors.

The PCOS also collected data on general and disease-specific measures of health-related quality of life, symptoms, comorbidity, and specific treatments received for prostate cancer using a mailed self-administered questionnaire. Most respondents completed the self-administered questionnaire (91%); those who did not return the questionnaire were contacted by telephone and asked to complete the survey by telephone or in person. Subjects were asked to recall their health-related quality of life and symptoms, including the domains of urinary, bowel, and sexual function, just before their prostate cancer was diagnosed. Demographic and socioeconomic questions from this survey were used to determine race/ethnicity, employment status, educational level, household income, insurance status, and marital status. A question assessing comorbidity asked about 12 medical conditions that were likely to affect prostate cancer treatment decisions and long-term quality of life. The conditions were derived from the Charlson index as well as the expert opinion of the PCOS investigators [12]. If the patient reported being told by a doctor that he had cerebrovascular disease, inflammatory bowel disease, liver disease, or ulcers, he received one point on his comorbidity score for each condition. If the patient reported that any of eight conditions – arthritis, diabetes, depression, hypertension, chest pain, heart attack, heart failure, or chronic lung disease – limited his activity or required prescription medications, he received 1 additional point for each of these conditions. In the analyses, comorbidity scores were divided into the categories of 0, 1, 2, and greater than or equal to 3 points.

We assigned screening status using information from the medical record abstract and the patient questionnaire. We considered men presenting with symptoms consistent with advanced prostate cancer, including bone pain, weight loss or hematuria, to be "clinically diagnosed." We initially created separate categories for men with only irritative or obstructive symptoms consistent with benign prostatic hyperplasia and an asymptomatic group who had neither prostate cancer nor lower urinary tract symptoms.

Clinical cancer stage was based on an algorithm using information abstracted from medical records. The algorithm was necessary because the community-based medical records were not detailed enough to classify cases by TNM (tumor, node, metastases) staging [13]. The algorithm defined T1 tumors as confined to the prostate with a normal digital rectal examination and no positive scans (magnetic resonance imaging, computed tomography, bone scan) or evidence of metastases. T2 tumors were defined as confined to the prostate, with abnormal or suspicious digital rectal examinations, but no positive scans or evidence of metastases. We defined clinically localized cancers as either T1 or T2 tumors. Initial treatment, based on medical record abstractions, was defined as treatment received within the first six months after diagnosis. We defined aggressive treatment as either radical prostatectomy or radiation therapy. We defined conservative management as androgen deprivation, either surgical or chemical, or watchful waiting.

### Statistical analyses

Descriptive statistics were calculated for ethnicity/race, age, stage at diagnosis, education, marital status, employment, income, digital rectal exam and PSA results, Gleason score from biopsy or transurethral resection of the prostate, comorbid conditions and self-reported general health status. We used contingency tables to compare men presenting without any symptoms, those with lower urinary tract symptoms alone, and those with prostate cancer symptoms. Although screening is defined as applying a diagnostic test to asymptomatic people [14], the prevalence of benign prostatic hyperplasia is very high among men at risk for prostate cancer [15]. We found that the men with only lower urinary tract symptoms were much more similar to asymptomatic men than to men we classified as having clinically diagnosed cancers. Therefore, we also considered cancers diagnosed in men who reported only lower urinary tract symptoms at the time of PSA testing to be "screening-detected." We used this combined screening-detected group to compare baseline characteristics against clinically diagnosed cases and in modeling treatment selection for clinically localized cancers. Logistic regression analyses were used to determine whether screening history was independently associated with selecting aggressive treatment versus conservative management among men with clinically localized prostate cancer. Covariates for this multivariate model, based on previous literature, included age, race/ethnicity, marital status, study site, education, insurance status, annual income, comorbidity, health status, and tumor characteristics [16,17]. We also examined interactions between screening status with age, comorbidity, PSA level, and Gleason score.

The results of the logistic regression models are shown as percentages receiving the treatment of interest, adjusting for the independent variables included in the model. These percentages were directly adjusted to the distribution of the variables among the weighted sample used in each model [18]. The probability of receiving the treatment of interest can then be directly compared across levels of the variables included in the model.

All analyses were performed with the Survey Data Analysis statistical package (Research Trial Institute, Research Triangle Park, North Carolina, 1997) to account for the complex survey design. We obtained unbiased estimates of parameters for all eligible prostate cancer patients in the PCOS areas by using the Horvitz-Thompson weight, which is the inverse of the sampling proportion for each sampling stratum (defined by age, race/ethnicity, and study area). A two-tailed P-value of < .05 was considered statistically significant.

## Results

The baseline demographic, socioeconomic, and clinical characteristics of the PCOS subjects are shown in Tables 1 and 2. The majority of subjects were non-Hispanic white men, older than sixty-five, and married at the time of diagnosis. Socioeconomic status was relatively high; a majority had more than a high school education, and a substantial proportion of subjects had private insurance. Among the study subjects, 10.7% presented with symptoms consistent with prostate cancer and were considered to be clinically diagnosed cases. Nearly two-thirds of subjects had lower urinary tract symptoms, while 30.9% were completely asymptomatic. Overall, 83.1% of men rated their general health at "good" or "excellent" before their cancer diagnosis.

We compared baseline characteristics of asymptomatic men, those with lower urinary tract symptoms alone, and men with clinically diagnosed cancers in Table 3. We found that the characteristics of men with lower urinary tract symptoms alone were closer to the asymptomatic men than to the clinically diagnosed cancer cases for race/ethnicity, socioeconomic status, health status, and cancer grade and stage. When we combined these two groups into a single category of screening-detected cases, we found significant differences between the screening-detected and clinically diagnosed cases. Men with screening-detected cancers were more likely to be non-Hispanic white, were younger age, and had a higher socioeconomic status. They also reported being healthier and were more likely to have early stage disease.

We then evaluated whether screening status independently predicted receiving aggressive treatment among the 2796 men who were diagnosed with clinically localized cancer. The primary treatment for these men was radical prostatectomy for 1535 (53.4%), while 518 (20.6%) underwent radiation therapy, 671 (26.0%) were treated conservatively; we had no treatment information for 72 subjects (2.5%). The results of the multivariate analysis are shown in Table 4. After adjusting for age, race/ethnicity, marital status, area of the country, education, insurance coverage, annual income, comorbidity, self-reported health status, and tumor characteristics, we found that men with screening-detected cancers were more likely to receive aggressive treatment. The adjusted percentage of men with screening-detected cancers undergoing aggressive treatment was 76% (95% CI 0.74, 0.78) vs. 70% (95% CI 0.64, 0.76), in men with clinically diagnosed cancers, OR = 1.5 (95% CI 1.1, 2.3), P = 0.05. Other factors that were significantly associated with aggressive treatment included geographic area, ethnicity, age, marital status, comorbidity, health status, and tumor characteristics. We found no significant interactions for treatment selection between screening status with age, comorbidity, PSA level, or Gleason score.

## Discussion

We found that the majority of cancers (89.3%) in a population-based PCOS cohort were detected by screening. Compared to men with clinically diagnosed prostate cancer, men with screening-detected cancers were younger, more likely to be married, less likely to be a member of a minority group, and in better health. The cancers detected by screening were more likely to be clinically localized and less likely to be poorly differentiated. Among men with clinically localized prostate cancers, those with screening-detected cancers were significantly more likely to undergo aggressive treatment, even after adjusting for demographics, comorbidity, and tumor characteristics

Our finding that a high proportion of prostate cancers diagnosed in 1994 and 1995 were detected by screening is consistent with the temporal correlation between the increased use of PSA testing and the increased incidence of prostate cancer in the USA beginning during the early 1990s [4,5]. Although prostate cancer incidence rates decreased for several years in the mid 1990s, more recent data show that incidence rates are again increasing [5,19,20] and survey results from the Centers for Disease Control's Behavioural Risk Factor Surveillance System (BRFSS) show that a high proportion of American men continue to undergo PSA testing [20]. These data suggest that our findings are still relevant for prostate cancers being diagnosed in the USA. We also found that men with screening detected cancers were more likely to have early stage cancers, again mirroring the epidemiologic data showing an increased incidence of early stage cancers and a decreased incidence of advanced stage cancers [4,5]. The majority of screening-detected tumors were moderately to poorly differentiated; however, a significantly higher proportion of clinically diagnosed cancers were poorly differentiated.

Previous data, including an analysis of the PCOS cohort, have shown African Americans to be twice as likely as non-Hispanic whites to present with advanced stage cancers [4,5,21]. In the current analysis, we found a greater prevalence of ethnic/racial minorities in the clinically diagnosed versus screening-detected cancers. This disparity may reflect ethnic/racial differences in accessing preventive health care services, particularly arising from socioeconomic barriers. This in turn could contribute to disparities in cancer stage at diagnosis [22-24]. However, African Americans also have been reported to demonstrate more skeptical attitudes towards screening [25] and the stage disparity could be due to racial differences in tumor aggressiveness [26].

Men with screening-detected clinically localized cancers were more likely to undergo aggressive treatment with radical prostatectomy or radiation therapy than men with clinically diagnosed cancers. The odds ratio for receiving aggressive treatment was statistically significant at 1.5, but the adjusted absolute difference between screening-detected and clinically diagnosed cases was only 6 percentage points. This modest association between screening status and treatment selection suggests that clinical practice may be only partly consistent with the American College of Physicians' view that "aggressive treatment is necessary to realize any benefit from the discovery of a tumor [27]." Our findings may reflect the scientific uncertainty about whether and how to treat screening-detected prostate cancers [28].

Our study has some potential limitations. We classified men presenting with symptoms of advanced cancer as being clinically diagnosed. We do not know that these symptoms actually prompted diagnostic PSA testing. However, the tumor registry medical record abstractors are trained to identify the events leading to a cancer diagnosis; they would attempt to record only symptoms consistent with cancer. Classifying PSA as a screening test is also difficult given the high prevalence of lower urinary tract symptoms in older men [15]. Few members of our study cohort were truly asymptomatic because nearly two-thirds reported lower urinary tract symptoms. However, our classifications for clinical diagnosis and screening detection were internally valid because men diagnosed with symptoms of advanced cancer were significantly more likely to present with advanced stage and more aggressive cancers than the combined group of men who were either asymptomatic or had only lower urinary tract symptoms. Additionally, when we compared demographic and socioeconomic characteristics across groups, we generally found that the men with lower urinary tract symptoms alone most closely resembled the asymptomatic men.

Selection bias may have occurred because 44% of the sampled patients did not complete the 6-month survey. Responders were younger than non-responders, more likely to be non-Hispanic white, had higher socioeconomic status, had earlier stage disease, and were more likely to receive radical prostatectomy. Results may be less generalizable to older men, those with lower socioeconomic status, or members of racial/ethnic groups other than non-Hispanic white. However, these were also the groups who were less likely to have screening-detected cancers. We do not believe that including these non-responders would have altered our findings on the differences between screening-detected and clinically diagnosed cancers. However, based on their demographics, socioeconomic status, and advanced disease stage, the non-responders were not likely to have a high proportion of screening-detected cancers and thus we may have overestimated the proportion of screening-detected cancers. Another potential limitation arose from asking subjects to recall their baseline symptoms 6 months after diagnosis. Recall errors could lead us to misclassify screening status. However, Legler and colleagues prospectively studied a subset of PCOS subjects and found high concordance for symptom recall at 6-months after diagnosis compared with reports at the time of diagnosis [29]. Finally, we may have had incomplete symptom data, particularly for questions appearing only in the medical record abstract. The abstracts would report a symptom if it appeared in the medical records; the absence of a symptom could be due to either the patient being asymptomatic or the physician's failure to ask about or record the symptom. We performed a sensitivity analysis by using only subject reported symptoms from the survey, then only symptoms reported on the medical record abstract, and then ultimately using a combination of both sources. The results for all analyses were essentially the same.

## Conclusion

The great majority of prostate cancers diagnosed in our study cohort were detected by screening. Appropriately, younger and healthier men were more likely to be diagnosed by screening. Minority status and lower socioeconomic status appeared to be screening barriers. Screening detected earlier stage and less histologically aggressive prostate cancers. After adjusting for baseline demographic, socioeconomic, clinical, and tumor factors, men with a screening-detected clinically localized cancer were slightly more likely to receive aggressive treatment, either radical prostatectomy or radiation therapy, than men with clinically diagnosed cancers.

## Competing interests

The author(s) declare that they have no competing interests.

## Authors' contributions

RMH and ALP initiated the project. SNS co-ordinated the data collection and was responsible for the data analyses. RMH, SNS, DE and ALP prepared the manuscript. All authors read and approved the final manuscript.

## Pre-publication history

The pre-publication history for this paper can be accessed here:



## Figures and Tables

**Table 1:** Baseline demographic and socioeconomic characteristics.

Variable	Number of subjects (Sample size = 3173)	Weighted percentages
SEER registry		
Atlanta	316	22.5
Connecticut	669	35.8
Los Angeles	938	13.5
New Mexico	342	11.5
Seattle	325	6.0
Utah	583	10.8
Ethnicity		
Non-Hispanic white	2187	75.7
Non-Hispanic black	539	13.8
Hispanic	447	10.5
Age		
< 49	102	2.3
50–64	1137	32.3
65–74	1336	44.5
75+	598	20.9
Current marital status		
Married	2499	78.6
Unmarried	637	20.3
Unknown	37	1.1
Education		
< High school degree	695	20.9
High school/some college	1419	43.6
≥ College degree	1014	34.0
Unknown	45	1.5
Insurance		
Private	2595	82.1
Public/Medicare	309	10.0
Unknown	269	7.9
Income (annual)		
< $20,000	792	23.3
$20 – 40,000	921	28.8
$40,000 +	1128	36.3
Unknown	332	11.6

Legend: SEER = Surveillance, Epidemiology, and End Results

**Table 2:** Baseline clinical characteristics.

Variable	Number of subjects (sample size = 3173)	Weighted percentages
Symptoms		
Asymptomatic	1001	30.9
Lower urinary alone	1832	58.4
Systemic	340	10.7
Comorbid conditions		
None	1211	37.2
1	1008	32.5
2	520	16.3
3+	434	13.9
Health status		
Excellent	534	16.9
Good	2104	66.2
Fair or poor	500	15.9
Unknown	35	1.0
PSA (ng/ml)		
<4	294	8.8
≥ 4	2675	84.5
Unknown	204	6.7
Digital rectal examination		
Abnormal	1741	54.8
Normal	1033	33.2
Unknown	399	12.0
Gleason score		
2 – 4	481	13.9
5 – 7	2029	65.1
8 – 10	373	11.6
Unknown	290	9.4
Tumor stage		
Local	2796	88.7
Regional	126	3.7
Advanced	251	7.6

Legend: PSA = prostate-specific antigen

**Table 3:** Distribution of baseline demographic, socioeconomic, and clinical characteristics by screening status.

Variable	Clinically diagnosed (weighted %)	Asymptomatic* (weighted %)	LUTS** (weighted %)	Asymptomatic or LUTS*** (weighted %)
SEER registry		P = 0.16*	P = 0.31**	P = 0.24***
Atlanta	14.2	14.2	12.9	13.3
Connecticut	19.1	24.7	21.9	22.9
Los Angeles	38.1	34.5	36.0	35.5
New Mexico	13.7	9.8	12.0	11.3
Seattle	4.3	5.9	6.4	6.2
Utah	10.5	10.9	10.8	6.8
Ethnicity		P < 0.01	P < 0.01	P < 0.01
Non-Hispanic white	65.1	79.9	75.5	77.0
Non-Hispanic black	20.8	12.4	13.3	13.0
Hispanic	14.1	7.8	11.2	10.0
Age		P < 0.01	P = 0.38	P ≤ 0.01
< 49	2.2	4.1	1.4	2.3
50–64	22.8	40.5	29.7	33.4
65–74	50.7	38.7	46.5	43.8
75+	24.4	16.7	22.4	20.5
Current marital status		P < 0.01	P = 0.03	P = 0.01
Married	72.9	81.4	79.7	80.3
Unmarried	27.1	18.6	20.3	19.7
Education		P < 0.01	P < 0.01	P < 0.01
< High school degree	33.3	16.2	21.7	19.8
High school/college	37.6	45.5	44.8	45.0
≥ College degree	29.7	38.3	33.5	35.2
Income (annual)		P < 0.01	P = 0.01	P < 0.01
< $20,000	36.8	19.0	28.4	25.1
$20 – 40,000	35.9	30.3	33.2	32.2
$40,000 +	27.3	50.7	38.4	42.7
Insurance		P < 0.01	P = 0.23	P = 0.01
Private	39.7	48.9	44.0	44.3
Public/Medicare	53.1	42.7	48.2	48.2
Unknown	6.4	8.4	7.9	8.1
Comorbid conditions		P ≤ 0.01	P = 0.41	P = 0.05
None	32.2	41.8	35.8	37.8
1	30.3	34.0	32.2	32.8
2	17.6	16.4	16.1	16.2
3+	19.8	7.8	16.0	13.2
Health status		P < 0.01	P < 0.01	P < 0.01
Excellent	9.9	23.0	15.2	17.9
Good	63.5	68.9	66.5	67.3
Fair or poor	26.6	8.1	18.3	14.8
PSA (ng/ml)		P = 0.01	P = 0.20	P = 0.05
<4	11.7	7.0	9.3	8.5
≥ 4	78.5	87.9	83.7	85.2
Unknown	9.8	5.1	7.0	6.4
Digital rectal examination		P = 0.10	P = 0.07	P = 0.06
Abnormal	59.4	54.0	54.3	54.2
Normal	27.0	34.0	33.9	33.9
Unknown	13.6	12.0	11.7	11.9
Gleason score		P < 0.01	P < 0.01	P < 0.01
2 – 4	9.6	13.6	14.9	14.4
5 – 7	53.4	69.9	64.6	66.5
8 – 10	22.3	8.3	11.4	10.3
Unknown	14.7	8.2	9.1	8.8
Tumor stage		P < 0.01	P < 0.01	P < 0.01
Local	74.6	92.8	89.1	90.4
Regional	4.0	3.2	4.0	3.7
Advanced	21.4	4.0	7.0	5.9

*P value comparing asymptomatic cases with clinically diagnosed cases.

** P value comparing lower urinary tract symptoms alone cases with clinically diagnosed cases.

***P value comparing asymptomatic and lower urinary tract symptoms alone cases with clinically diagnosed cases.

Legend: LUTS = lower urinary tract symptoms, SEER = Surveillance, Epidemiology, and End Results; PSA = prostate-specific antigen

**Table 4:** Multivariate model of factors associated with undergoing aggressive^a ^treatment for clinically localized cancer (n = 2796).

Variable	Received aggressive treatment		Wald F P-value
	Adjusted percentages (95% CI)	Odds ratio (95% CI)	

Screening history			0.05
Not-screened	76 (74, 78)	1.0	
Screened	70 (64, 76)	1.5 (1.1 – 2.3)	
SEER registry			< 0.01
Los Angeles	71 (67, 75)	1.0	
Atlanta	89 (85, 93)	4.8 (2.7 – 8.5)	
Connecticut	77 (73, 81)	1.5 (1.1 – 2.2)	
New Mexico	72 (66, 78)	1.1 (0.7 – 1.7)	
Seattle	73 (67, 79)	1.2 (0.8 – 1.8)	
Utah	77 (73, 81)	1.5 (1.0 – 2.3)	
Ethnicity			< 0.01
Non-Hispanic white	76 (74, 78)	1.0	
Non-Hispanic black	69 (63, 75)	0.6 (0.4 – 0.9)	
Hispanic	79 (75, 83)	1.2 (0.8 – 1.9)	
Age			< 0.01
< 49	95 (92, 100)	1.0	
50–64	89 (87, 91)	0.4 (0.2 – 1.2)	
65–74	79 (77, 81)	0.2 (0.1 – 0.5)	
75+	41 (35, 47)	0.03 (0.01 – 0.08)	
Current marital status			< 0.01
Married	77 (75, 79)	1.0	
Unmarried	71 (67, 75)	0.6 (0.5 – 0.8)	
Education			0.95
< High school degree	76 (72, 80)	1.0	
High school/college	76 (74, 78)	1.0 (0.7 – 1.3)	
College degree	76 (72, 80)	0.9 (0.7 – 1.4)	
Insurance			0.25
Private	76 (74, 78)	1.0	
Medicare/Public	74 (68, 80)	0.9 (0.6 – 1.4)	
Unknown	81 (75, 87)	1.6 (0.9 – 2.8)	
Income (annual)			0.98
< $20,000	76 (72, 80)	1.0	
$20 – 40,000	76 (72, 80)	1.0 (0.7 – 1.4)	
$40,000 +	76 (72, 80)	1.0 (0.7 – 1.5)	
Comorbid conditions			0.01
None	78 (74, 82)	1.0	
1	77 (73, 81)	0.9 (0.7 – 1.2)	
2	77 (73, 81)	0.9 (0.6 – 1.3)	
3+	68 (62, 74)	0.5 (0.3 – 0.7)	
Health status			< 0.01
Excellent	80 (76, 84)	1.0	
Good	77 (75, 79)	0.8 (0.5 – 1.2)	
Fair or poor	67 (61, 73)	0.4 (0.3 – 0.7)	
PSA (ng/ml)			< 0.01
<4	69 (63, 75)	1.0	
≥ 4	77 (75, 79)	1.7 (1.1 – 2.6)	
Unknown	68 (60, 76)	0.9 (0.5 – 1.9)	
Gleason score			< 0.01
2 – 4	68 (62, 74)	1.0	
5 – 7	78 (76, 80)	2.0 (1.4 – 2.8)	
8 – 10	71 (65, 77)	1.2 (0.7 – 2.0)	
Unknown	80 (74, 86)	2.3 (1.3 – 4.2)	

^a^Aggressive treatment was defined as radical prostatectomy or radiation therapy.

Legend: SEER = Surveillance, Epidemiology, and End Results; PSA = prostate-specific antigen
